# Analyzing the Caloric Variability of Bites in a Semi-Naturalistic Dietary Setting

**DOI:** 10.3390/nu17132192

**Published:** 2025-06-30

**Authors:** Mohammad Junayed Bhuyan, Luca Vedovelli, Corrado Lanera, Daniele Gasparini, Paola Berchialla, Ileana Baldi, Dario Gregori

**Affiliations:** 1 Unit of Biostatistics, Epidemiology and Public Health, Department of Cardiac, Thoracic, Vascular Sciences and Public Health, University of Padova, 35131 Padova, Italy; mohammad.bhuyan@ubep.unipd.it (M.J.B.); luca.vedovelli@ubep.unipd.it (L.V.); corrado.lanera@ubep.unipd.it (C.L.); daniele.gasparini@ubep.unipd.it (D.G.); ileana.baldi@unipd.it (I.B.); 2 Department of Clinical and Biological Sciences, University of Torino, 10043 Orbassano, Italy; paola.berchialla@unito.it

**Keywords:** bites, healthwear, caloric intake, food portion size effect, streaming data

## Abstract

**Background:** Obesity is a major public health issue in developed countries, primarily managed through dietary interventions and physical activity. Food portion sizes influence the estimation of energy intake, particularly through bites, of which characteristics remain insufficiently defined. This study investigates the variability in bite energy content. **Methods:** This observational study was conducted over 14 months. Thirteen types of packaged food were provided to 30 Italian healthy volunteers (mean age 26.8 ± 8.5 years) in a semi-naturalistic dietary feeding setting. Participants’ anthropometric measurements were recorded. A total of 1850 bites were weighed and 420 bites were assessed for volume and energy content. **Results:** Bite volume and mass explained bite energy content at different rates. The most influential anthropometric feature was waist circumference. Gender modified the association between waist circumference and bite characteristics; males showed increased bite volume, mass, and energy content as waist circumference increased, whereas females showed little or no association. Age was inversely associated with bite volume and mass, with younger participants having larger bites. Gender significantly influenced average bite size, with females showing lower values than males. The use of a fork was associated with higher bite volume, mass, and energy compared to a spoon. Food eaten with bare hands had lower mass but higher energy content compared to food eaten with a spoon. The variability in bite energy was considerably greater per bite than per gram, reflecting the combined influence of food texture, bite size, and cutlery used. **Conclusions:** Bite energy variability, influenced by intrinsic factors (gender, age, waist circumference) and extrinsic factors (cutlery, food texture), significantly impacts portion size effect. Future bite counters should consider these elements for accurate dietary assessment.

## 1. Introduction

Obesity is one of the most pressing public health concerns in developed countries, primarily resulting from an unbalanced diet and sedentary lifestyle. Although multifactorial, its primary determinant is excessive caloric intake [1]. The forerunner condition of obesity is being overweight, which affects 43% of aged people worldwide [2]. Moreover, obesity is strictly correlated to cardiovascular diseases, respiratory pathologies, cancer and diabetes [3,4], and it is a life-threatening condition that should be continuously monitored since its complications are what the WHO defines as silent killers [5]. Nowadays, obesity treatments envisage a reduction in the caloric intake together with a change in one’s lifestyle, especially including a right amount of physical activity [6]; however, it is essential to monitor these two components, particularly regarding the caloric intake; in fact, patients are urged to keep a diary of what they eat, and food registers, food diaries, and food frequency questionnaires have been the usual tools available to monitor the caloric intake [7,8]. Besides these basic methods, a better knowledge of the composition of the food offered by large distribution chains is often supported by compulsory labeling [9]; yet, that information is usually estimated and can lead to errors in the caloric amount count [10], mainly because it is left to self-monitoring, which generally leads to biased estimates [11,12,13]. Moreover, the human factor in estimating must be thoroughly considered: people are more likely to underestimate the caloric content of the main dishes and to choose higher-calorie side dishes, drinks, or desserts when fast-food restaurants advertise to serve healthy food [14]. Beyond merely psychological factors, even gender resulted in being an affecting element: females would tend to overestimate food caloric content by 19% [15]. Moreover, other biasing factors include body mass index (BMI), portions size, diet history, and the kind of food. In addition to portion size, bite size has also been shown to significantly influence calorie estimation accuracy. In a randomized crossover trial of 44 adults consuming varying portion sizes of pasta, faster eating rates, and larger bite sizes—despite low within-person variability—were significantly associated with increased food intake across all meals [16].

More recently, to reduce the inaccuracy due to self-monitoring, alternative methods belonging to healthwear have been proposed to provide a real-time, continuous, and precise identification of eating episodes, classification of foods, and monitoring of the caloric intake [17,18,19]. Healthwear aims to develop wearable devices that can continuously monitor patients and subjects’ vitals, and so, wearable devices have been designed to monitor either the caloric expenditure or the caloric intake itself. The former was abandoned because of the high rates of measurements’ errors [20,21], and nowadays many have been researching whether it is possible to calculate the caloric intake and to help patients and subjects have an automatic, auto-filling, diet diary to address the obesity problem consistently. The methodology used usually relies on two assumptions: (a) using the bite as a unit of measurement of the caloric intake, and (b) tracking the movements of the whole or of a part of the upper limb since in healthy subjects, it is the part of the body that actually brings the food to the mouth, composing what a bite actually is. Eleven wearable devices, mostly consisting of wrist-worn accelerometers and with an algorithm embedded, have been developed to count the bites people make while eating [22] so that caloric intake. So far, results have shown that bite count and the energy intake were positively correlated, with an average per-individual correlation of 0.53 [23]. Moreover, bite-counting devices were better than subjects in estimating the caloric intake; the sensitivity of these devices is equal to 75%, with a positive predictive value of 89% [24]. Finally, the estimation could be improved by calibrating the device to one’s bite size and/or to the caloric amount that bites have on average. Hence, the importance of investigating the variability in the bites regarding size and energy content, and the potential influence on the bites themselves by factors such as gender and BMI is a relevant scientific issue. Many are the flaws in these studies, first of all, due to the fact that they tend to analyze eating in conditioned environments, with many psychological implications [25,26].

Since the bites have been confirmed to be an optimal choice of unit of measurement [27], one of the biggest challenges is to analyze what can affect the caloric content of each bite. Therefore, this report aims to study the variability in the energy content in the bites generated by eating packaged food items to understand whether and how it might affect the estimation of the caloric intake and which factors might condition the variability itself.

## 2. Materials and Methods

This study is based on data from the NOTION study (acronym of ‘measuriNg calOric inTake at populatION level’). This research project’s main aim was to estimate the caloric intake through the definition of an algorithm embedded in a wearable device worn at the wrist. The Bioethics Committee of the University of Torino (Italy), acting as the Institutional Review Board, approved the study protocol on 12 July 2017, number 256091 [28].

### 2.1. Experimental Strategy

With the aim to analyze bites and their features (mass, volume, energy content), the experimental design of the NOTION considered the following three main sources of bias, mostly coming from the achievements in the literature: (a) morphometric and clinical individual features (gender, age, nutritional status, etc.); (b) food characteristics (food texture, composition, hardness, caloric content, etc.); and (c) spontaneity in eating movements, depending on conditioning due to public/private place, conventional rules, time for meals, etc.

To reduce these biases, subjects were all Italian, 20-to-30-year-old people, whose individual features were gathered to act, where appropriate, as confounding factors. Moreover, food items were standardized by using standard portions of manufactured food with known biochemical and energetic composition. Finally, to ensure a free-living environment for the meals, the experimental phase was held in an authorized public catering area—a cafeteria generally attended by students and University workers.

**Figure 1:**
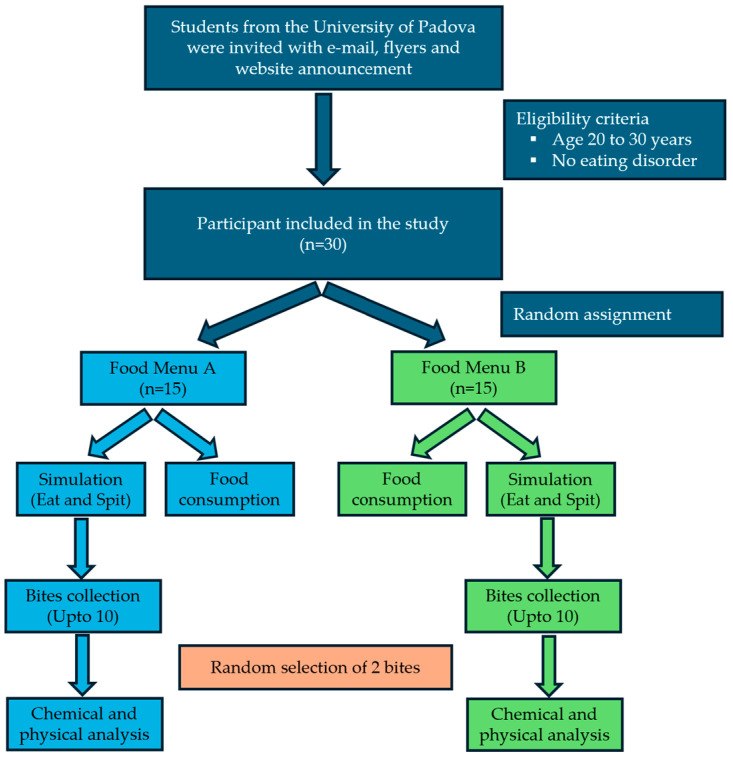
NOTION study design and flow-chart of its steps.

To measure the bite features for each subject, the eating activity was divided into two steps. In the first step (or fake eating) up to 10 spat bites for each food were collected; in the second step (or actual meal), subjects were video recorded while they were fully completing the served food (Figure 1). Both steps included the recording of the movements of the wrist while eating.

### 2.2. Sample Population and Experimental Phase

The data collection took place in a cafeteria near the Laboratory of Dietetics and Nutraceutical Research of the Department of Cardiac, Thoracic, and Vascular Sciences, from October 2017 to January 2018. A total of 30 healthy subjects were recruited on a voluntary basis, among which were 14 males (age 26.6 ± 10.3 years and BMI 23.6 ± 2.7 Kg/m^2^) and 16 females (age 26.9 ± 6.9 years and BMI 21.8 ± 1.8 Kg/m^2^). The subjects included were the twenty of the NOTION study, plus the ten of the pilot phase. The participant recruitment strategies for the NOTION study consisted of social media advertisements, like Facebook and the official web page of the DCTVPH, and via flyers. People interested in taking part, once informed about the aims and the methods of the study and if they fulfilled the inclusion criteria, signed their informed consent. The 30 subjects underwent a clinical visit, during which several anthropometric measurements were collected or calculated. A dietician measured subjects’ weight and height using a SECA 220 weighting scale (SECA, Hamburg, Germany) with a stadiometer (maximum capacity 220 Kg; accuracy: 1 hg; 1 mm), and the BMI was derived. Waist and hip circumferences were measured using a tape measure (accuracy: 1 mm), and they were divided to achieve the Waist–Hip Ratio (WHR). Four skinfolds were measured, using a Holtain 610 Caliper (accuracy: 0.1 mm, (Holtain Ltd., Crosswell, United Kingdom); biceps (BS), triceps (TS), scapular (SS), and pelvic (PS) skinfolds were then combined to calculate body density to derive the Fat Mass (FM) and the Free Fat Mass (FFM), using Durning & Womersley [29] and Siri-Brozek [30] equations. Their ages and intensity of physical activity were asked in a questionnaire about diet and lifestyle habits.

Dieticians chose the food to serve to simulate a complete Italian daily diet. Each of the two menus was composed of 7 different types of pre-packaged food items, organized in breakfast, lunch, snack, and dinner. Subjects were randomly assigned one of the two menus (Table 1). Volunteers were summoned on two different days at fixed times, to have breakfast (7:30 a.m.) and lunch (11:30 a.m.) on the first day, and snack (7:30 a.m.) and dinner (11:30 a.m.) on the second day at an authorized public catering area.

### 2.3. Food Collection and Analysis

In the simulated fake eating step, each volunteer brought the food to the mouth without swallowing it: each bite was then spat into a plastic bag previously labeled with a unique alphanumeric code; the code was constructed to precisely identify the subject, the meal, the food item, and the number of bites. The maximum number of bites collected for each food item and each subject was 10; other potential bites were not collected, and the subjects were suggested to stop fake-eating. During the step of actual eating, participants were asked to eat the meal continuously and naturally; meanwhile, all the bites collected were conveyed to the Laboratory of Dietetics and Nutraceutical Research in a refrigerated bag. Each bite was weighed using the Gibertini Crystal 100 scale. Two bites of each food item were randomly selected to be measured in terms of volume (mL), with an A-class, 250 mL, graduated cylinder, and energy content (Kcal) with the IKA C200 bomb calorimeter, for a total of 420 measurements. Twenty of these (5%) were excluded from these surveys because of analytical problems. Before the beginning of the project, bomb calorimeter calibrations were performed using known amounts of benzoic acid as the standard. The values obtained were within 1% of the combustion heat known for benzoic acid, confirming the accuracy and reliability of the instruments.

### 2.4. Sample Preparation for Bomb Calorimetry

For the calorimetric analysis, each bite was well homogenized, using the mortar for low-fibrous foods (such as chicken meatballs with tomatoes or mozzarella), or the laboratory grinder for foods that are harder to homogenize (such as chicken and artichokes or eggplant parmigiana). Only for liquid food (soup), samples were dried in a 60 °C oven for 4 h, before being analyzed, to reduce a possible interference of the water on the sample caloric value. The result of the analysis was established considering the weight of the wet sample.

After homogenization of each bite, a rate ranging between 0.3 and 0.5 g of sample was introduced into the calorimetric bomb. The amount was established based on the presumed caloric content of each food item. The sample was placed inside a steel container (the bomb), which was filled with oxygen (O_2_) up to high pressure. The energy content of the sample (S, in Joule) is directly calculated by the instrument as follows: S = (W × ΔT)/m where W is the work made by the system (J), ΔT (K) is the difference between the beginning temperature and the final one, and m is the exact weight of sample (g). The values were then converted into calories/grams [31].

Although the reaction happening in the bomb calorimeter simulates what happens in the human body, it is necessary to consider that ingested macronutrients may not be made fully available to tissues, and the tissues themselves may not be able to oxidize substrates made available to them entirely. To correct for incomplete digestibility, the corresponding metabolizable energy values were calculated by subtracting 1.25 kcal per gram of protein from the gross energy [32].

### 2.5. Statistical Method

Suitable descriptive analyses were applied to subjects’ and bites data. Mean values and standard deviation were used to summarize quantitative variables.

The variability in the bite energy amount, both per gram and the whole bite, was evaluated by means of the coefficient of variation (CV%). Ninety-five percent confidence intervals (95%CI) were estimated by means of the non-parametric bootstrap method [33].

To explore the factors associated with bite characteristics, simple linear Bravais-Pearson’s correlation coefficients were computed among subjects’ anthropometric data and bite volume, mass, and energy, both on the whole sample and by gender. The analysis of covariance (ANCOVA) was applied to evaluate the role of the modifier for sex on the relationship between anthropometry and the bite characteristics. Considering that anthropometric indices were highly correlated among themselves, in the statistical analysis, only waist circumference was included as a covariate, since it showed the highest simple correlation with bite features. In the statistical analysis, food items were used as listed or categorized based on cutlery (fork, spoon, bare hands).

In evaluating the effects of covariates on bite features, the design effect was applied considering bites clustered at the level of the type of food. The volume, mass, and energy content of a bite was evaluated using separate multiple mixed regression models with restricted maximum likelihood, in which covariates were fixed effects and foods and cutlery were random effects. The variance was accounted by including food (or cutlery) as a random categorical variable in the models. Given the interaction due to sex on the relationships between anthropometry and the bite characteristics, the multivariable models were built stratifying data by sex.

The regression models explaining the bite volume (Model 1) included age, waist circumference, and food as covariates; for mass (Model 2), they included age, volume, and food. On the other hand, bite energy content was described by means of four different models, including various factors as covariates as follows: age, volume, mass, and food for Model 3; age, volume, mass, and cutlery for Model 4; age, waist circumference, and type of food for Model 5; and age, waist circumference, and cutlery for Model 6. In the models, covariates were included as fixed effect, apart from food and cutlery.

To estimate the goodness to fit of the model, the R^2^ for the corresponding fixed-effects model was used.

The significant level was tested at 0.05 for two-tailed tests. All the statistical analyses and the mathematical models were performed using R v.4.1 [34].

## 3. Results

Overall, 1850 bites were collected during the fake-eating phase and weighed. Among them, for 420 bites, volume and energy content were measured, corresponding to 30 bites for each food item, except for yogurt, for which 60 bites were analyzed. A total of 20 bites out of 450 (5%) were excluded because of analytical problems.

The mean ± SD of volume (mL), mass (g), and energy content (Kcal) per bite for all 400 samples are, respectively, 20.0 mL ± 8.1 mL; 11.4 g ± 6.7 g; and 21.5 Kcal ± 14.4 Kcal. These statistics were also calculated by food items, by gender, by meal, and by cutlery (Table 2).

The results indicate substantial variability in bite dimensions across different food items. The smallest bite volume was observed in vegetable soup (V = 13.8 mL), whereas the largest was found in tagliolini with mushrooms (V = 28.9 mL). Similarly, the lowest bite mass was recorded for rusks and jam (m = 3.5 g), while the highest was for tagliolini with mushrooms (m = 20.3 g). Energy was minimum for rusks and jam (E = 6.1 Kcal/bite) and maximum for tagliolini with mushrooms (E = 39.0 Kcal/bite). Moreover, as expected, gender was significantly associated with the mean dimension of bites, showing lower values for females than for males. Meal energy content of bites in the Italian-style diet of this research proved to be significantly higher at lunch and at snack time (27.8 Kcal/bite and 28.8 Kcal/bite, respectively) than at breakfast or dinner (13.0 Kcal/bite and 20.1 Kcal/bite, respectively). In addition, the use of cutlery influenced the features of the bites. When a fork was used to eat the food, bites showed higher volume, mass, and energy than when using a spoon (volume: 20.7 mL vs. 15.2 mL; mass 14.3 g vs. 11.8 g; energy: 25.8 Kcal/bite vs. 8.5 Kcal/bite); meanwhile, bare-hand-eaten-food showed lower mass (5.8 g) and higher energy (25.8 kcal/bite) than when using a spoon.

**Figure 2:**
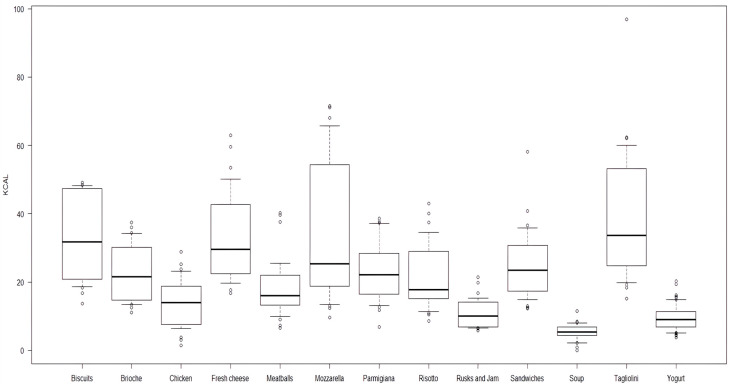
Box-plot of bite energy content, expressed in Kcal/bite, by each food item.

Focusing on the energy content per bite, the variability in each food item is shown in Figure 2. As shown, a noticeable variability was seen in the distribution for food such as tagliolini with mushrooms, mozzarella, biscuits, and fresh Italian cheese. Furthermore, the CV% and 95%CIs are shown in Table 3.

We calculated the CV% for energy content per gram (Kcal/g) of food to emphasize that the texture and the heterogeneity of the food matrix may affect the energy content of the bite. Data showed minimum CV% values for homogeneous food such as brioche [1.8% (1.6%; 2.1%)], and maximum values for heterogeneous food such as chicken and artichokes [41.2% (35.4%; 48.4%)].

The CV% of the energy content per bite (Kcal/bite) was calculated to take into account the additional relevant variability due to the size of the bite. In Table 3, values for CV% of the energy content per bite by gender, meals, and the type of cutlery were also shown.

**Figure 3:**
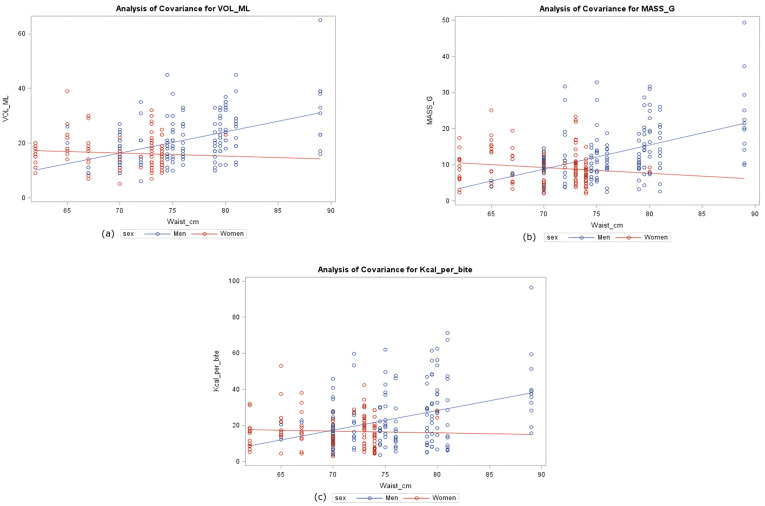
Results from covariance analysis. Volume (**a**), Mass (**b**), and Energy (**c**) of bites were described by sex and waist circumference in multiple separate models. The role of sex as a modifier of the effect caused by waist was tested.

To appreciate the potential role of anthropometric characteristics on the bite size, the covariance analysis is shown in Figure 3. Volume, mass, and energy of the bites were described by gender and waist in multiple separate models. The analysis emphasizes that gender has a role in modifying the effect of the waist on the considered bite characteristics; moreover, it shows that the same gender-depending behavior is constant for different variables.

The results from the regression mixed models are shown in Table 4. For what concerns the energy content per bite, Model 3, for both sexes, explained about 90% of the variability, whereas Model 5, where cutlery replaces the type of food as a covariate, showed a goodness of fit drop to about 65%.

## 4. Discussion

### 4.1. Gender and Cutlery in Affecting Bite Dimensions

Measuring caloric intake through bite analysis requires a thorough understanding of the factors influencing bite characteristics. Our findings indicate that bite energy content exhibits significant variability, influenced by factors such as bite volume, bite mass, and food type. Gender was found not only to be a determinant of volume and mass but also a modifier in the relationships between waist circumference and bite volume and between age and bite mass.

The expected differences between bite characteristics by gender—that is, males made bigger bites than females—is in accordance with several research that observed gender differences in single-food laboratory studies [35,36,37]. The analysis of covariance showed that gender modified the effect of the waist circumference on the characteristics of the bites. Indeed, as the waist grew, males made bites with higher volume, mass and energy content, while in females, waist circumference showed lighter or no association with bite features. In the present study, gender function might be linked to gender-specific esthetic, behavioral, and educational attitudes among young adults; hence, the generalizability of this result requires confirmatory studies that include subjects of a wider age range. On the other hand, the type of cutlery used to eat was found to be associated with the physical characteristics of the bites and therefore to their energy content; in fact, a spoon provides a standard size of a bite correlated with its size. Thus, there is a low variability in bite energy content for food such as yogurt and soup. Eating a portion of food, instead, with bare hands or a fork and a knife result in bites with different sizes according to the individual and with different energy content: this would concern food such as biscuits, mozzarella, and fresh Italian cheese.

### 4.2. Food Texture and Bite Energy Content Variability

Another primary factor influencing the caloric variability between bites is the type of food in terms of composition. The food that is heterogeneous in the composition of the ingredients showed high variability between grams of the same food; moreover, we must also consider the tendency of each volunteer to eat each component of a dish separately or together, thus adding this individual variability to the heterogeneity of the food. In fact, this unconscious choice can influence the bite composition, leading to a higher or lower caloric content, which would partially explain the variability observed between the bites of the same food; in fact, when the subjects tended to eat the different components of each dish separately, the bomb calorimeter returned different values than the ones of the labels.

A factor that could potentially bias the caloric measurement per bite relies on the fibrous component of the food: the bomb calorimeter sometimes burned food incompletely, thus distorting the caloric esteem. This concern might be applied to food items like chicken and artichokes, eggplant parmigiana, and tagliolini with mushrooms. Heterogeneity and fibrous components are the source of variability in the energy content per gram of food, ranging, in the present research, from 1.9% (brioche) to 41.2% (chicken and artichokes), while the variability per bite was much higher, better reflecting the variability in bite dimensions (volume and mass). This result suggests that the variability in the energy content per bite is affected by bite volume and mass, much more than by inhomogeneity in food texture.

### 4.3. In-Meals Differences

Regarding the general aspects of the study, the analysis of the meals shows that snack is the meal that brings the highest caloric content per bite, with an average of 28 Kcal/bite. Although it could seem tautological, especially considering the food choices (Menus A and B), Italian nutritional habits have been changing towards packaged food, mostly snacks rather than regular meals, contributing to their diffusion, which often leads to underestimating their high caloric load. This aspect is even more serious considering that the consumption of snacks by children has been steadily increasing in recent years and the trend is growing [38,39]. The adoption of packaged food for snacks means smaller portions of highly caloric food, with bites quite homogeneous in their energy content (brioche CV% = 35.1%, biscuits CV% = 38.5%, sandwich CV% = 34.9%).

### 4.4. Models and Surrogate Variables

Theoretically, bite characteristics are determined, sequentially, by bite volume, which, in turn, conditions bite mass; yet mass is expected to be the major contributor to the variability in the energy content of the bites. Similarly, the type of food should affect bite volume, mass, and energy content. Consequently, to accurately estimate the caloric intake of the meals by using bites, the detection of factors explaining the volume variability is crucial. In this study, all these expected relationships have been confirmed by the results of the regression models.

As suggested by the literature [40,41], in this statistical analysis anthropometry was taken into account in the building of the models concerning bite volume. The anthropometric indexes were all strictly correlated to each other; among them, waist circumference was shown to be the best contributor in explaining bite volume, both in females and in males.

The results from the regression models showed that, if volume, mass, and type of food were accurately measured, about 90% of bite energy content variability would be explained. However, if volume and mass were unknown, assuming the type of food as known and using age and gender and surrogate variables, models would explain the same variability of up to 48% for females and 71% for males. If the type of cutlery substituted the type of food, the resulting relationships would be useless: 24% for females and 36% for males.

The results from this study might be limited by the fact that other not-investigated factors might act as biases of the caloric intake at meals. For instance, using a bite-counter device to slow the bite-rate may lead to a reduction in the energy intake for individuals who consume higher amounts of food [42]. Moreover, other studies showed that people consume more food when the portion size is larger, and therefore the caloric intake is higher [16,43]; in fact, using standard food portions, the size effect on the caloric intake could not be detected.

### 4.5. Semi-Naturalistic vs. Real Life

Finally, in this experimental study, it was possible to accurately measure bite characteristics in a semi-naturalistic setting, building multiple regression models to explain the actual caloric values of the bites as provided by the bomb calorimeter. However, generally, bite measurements and type of food are unknown, and they should be estimated using setting variables (sex, age, waist circumference) and, mostly, detecting bites and the type of food from wrist movements.

### 4.6. Clinical and Practical Implications and Future Perspectives

The ability to predict caloric intake based on bite characteristics creates opportunities for the development of personalized dietary monitoring tools, particularly in the context of wearable health technologies. The integration of bite-size data, stratified by sex and anthropometry, could significantly improve the accuracy of caloric estimation in free-living conditions, supporting obesity prevention strategies, weight loss programs, and nutritional counseling. Furthermore, understanding how variables such as cutlery and food texture influence intake can guide dietary behavior interventions.

Future research should explore the generalizability of these models in broader populations, including children, older adults, and individuals with eating disorders or chronic conditions. Additionally, studies should investigate the feasibility of incorporating real-time bite characterization into wearable devices, expanding from laboratory settings to fully naturalistic environments such as homes or restaurants. Machine learning algorithms trained on multimodal sensor data could further refine energy intake estimation and help automate self-monitoring in digital nutrition platforms.

## 5. Conclusions

This study provides insights on how bite variability affects caloric intake estimation in adults. Our findings confirm that bites represent a promising unit for estimating overall caloric intake during meals. Moreover, the results show that several factors, like age, sex, and waist circumference, influence bite variability. To monitor one’s daily caloric intake accurately, a bite-counter could be developed considering all these aspects. Nevertheless, it will be necessary to identify other factors influencing bite variability to increase the accuracy in the caloric content determination. These findings support the development of advanced wearable systems that integrate individual anthropometric data and food-type classification for real-time caloric monitoring. Future research should explore multimodal sensor fusion and machine learning algorithms to enhance accuracy and enable deployment in diverse, uncontrolled eating environments.

## Figures and Tables

**Table 1:** Composition of the two menus (A, B) randomly assigned to the 30 volunteers recruited in the NOTION study.

Meal	Menu A	Kcal/Portion
Breakfast	Rusks and jam	114 Kcal/41 g
	Yogurt	186 Kcal/170 g
Lunch	* Risotto * with asparagus	471 Kcal/300 g
	*Mozzarella*	242 Kcal/100 g
Snack	Biscuits	278 Kcal/55 g
Dinner	Vegetable soup	135 Kcal/310 g
	Artichoke chicken	199 Kcal/120 g
**Meal**	**Menu B**	**Kcal/Portion**
Breakfast	Brioche	99 Kcal/28 g
	Yogurt	186 Kcal/170 g
Lunch	*Tagliolini* with mushrooms	546 Kcal/300 g
	Chicken meatballs with tomato sauce	135 Kcal/120 g
Snack	Sandwich	195 Kcal/70 g
Dinner	Eggplant *parmigiana*	309 Kcal/300 g
	Italian fresh cheese	269 Kcal/100 g

**Table 2:** Physical and chemical features per bite for the whole sample of collected bites and divided by food item, by gender, by meal, and by cutlery.

	N of Bites	VOLUME (mL)	MASS (g)	ENERGY (Kcal)
		Mean ± SD [Median]	Mean ± SD [Median]	Mean ± SD [Median]
Whole Sample	400	20.0 ± 8.1 [19.0]	11.4 ± 6.7 [10.0]	21.5 ± 14.4 [17.8]
*by* Food Item				
Rusks and jam	30	19.1 ± 5. [19.0]	3.5 ± 1.4 [3.3]	10.8 ± 4.7 [10.0]
Yogurt	58	15.7 ± 5.2 [15.0]	11.6 ± 3.7 [10.7]	9.4 ± 3.7 [8.9]
Brioche	28	25.0 ± 8.5 [24.0]	6.4 ± 2.2 [6.3]	22.8 ± 8.0 [21.5]
*Risotto* with asparagus	30	18.7 ± 5.7 [17.0]	13.3 ± 5.4 [11.6]	21.6 ± 6.0 [17.6]
*Tagliolini* with mushrooms	30	28.9 ± 10.7 [29.0]	20.3 ± 9.1 [17.0]	39.0 ± 18.0 [33.6]
*Mozzarella*	30	19.6 ± 10.4 [16.5]	12.8 ± 8.3 [10.1]	32.2 ± 19.7 [22.5]
Chicken meatballs with sauce	30	19.0 ± 6.9 [18.0]	12.4 ± 5.2 [11.2]	18.2 ± 8.5 [16.0]
Biscuits	30	22.7 ± 7.7 [22.0]	6.6 ± 2.6 [6.0]	33.6 ± 12.9 [31.7]
Sandwiches	24	21.9 ±6.1 [21.0]	7.1 ± 2.2 [6.9]	22.9 ± 8.0 [21.0]
Vegetable soup	20	13.8 ± 3.7 [12.5]	12.2 ± 2.4 [12.1]	6.1 ± 2.0 [5.9]
Artichoke chicken	30	19.5 ± 6.8 [18.5]	10.9 ± 4.1 [9.3]	14.0 ± 7.0 [13.8]
Eggplant *parmigiana*	30	23.1 ± 8.3 [21.5]	19.1 ± 6.9 [18.2]	22.7 ± 8.4 [22.1]
Italian fresh cheese	30	16.3 ± 5.1 [16.0]	11.4 ± 4.6 [10.7]	32.8 ± 12.8 [29.5]
*by* Gender				
Female	208	18.3 ± 6.9 [17.0]	9.8 ± 5.4 [8.7]	18.4 ± 11.2 [16.2]
Male	192	21.9 ± 8.9 [21.0]	13.3 ± 7.5 [11.3]	24.9 ± 16.5 [20.3]
*by* Meal				
Breakfast	116	18.8 ± 8.0 [18.0]	8.3 ± 4.6 [8.2]	13.0 ± 7.6 [11.0]
Lunch	120	21.6 ± 9.6 [19.0]	14.7 ± 7.8 [12.3]	27.8 ± 16.8 [22.2]
Sneak	54	22.4 ± 7.0 [21.0]	6.8 ± 2.4 [6.5]	28.8 ± 12.1 [24.9]
Dinner	110	18.6 ± 7.2 [17.0]	13.5 ± 6.0 [12.2]	20.1 ± 13.0 [17.6]
*by* Cutlery				
Fork	210	20.7 ± 8.7 [19.0]	14.3 ± 7.3 [12.5]	25.8 ± 15.2 [21.9]
Spoon	78	15.2 ± 4.9 [15.0]	11.8 ± 3.4 [11.3]	8.5 ± 3.7 [8.1]
Hand	112	22.1 ± 7.3 [21.0]	5.8 ± 2.5 [5.3]	22.5 ± 12.1 [20.2]

**Table 3:** Coefficient variation (CV%) and 95% CI for bite energy values, estimated by a bootstrap non-parametric procedure, divided per gram and per bite, and by food items, by gender, by cutlery, and by meal.

		CV% Kcal/g	95%CI	CV% Kcal/Bite	95%CI
Food Items	Rusks and jam	10.0	*6.9*, *13.2*	38.5	*35.1*, *43.5*
	Yogurt	19.4	*16.0*, *23.5*	39.4	*34.8*, *45.3*
	Brioche	1.9	*1.6*, *2.2*	35.1	*32.3*, *39.4*
	*Risotto* with asparagus	9.9	*8.8*, *11.4*	44.2	*41.0*, *49.0*
	*Tagliolini* mushrooms	8.0	*8.6*, *11.2*	46.2	*40.5*, *53.6*
	*Mozzarella*	9.6	*15.5*, *18.4*	61.1	*57.4*, *66.9*
	Chicken meatballs sauce	6.8	*12.5*, *15.0*	46.6	*42.0*, *54.1*
	Biscuits	4.9	*3.1*, *7.5*	38.5	*35.5*, *42.9*
	Sandwiches	16.5	*15.0*, *18.8*	34.9	*32.2*, *39.3*
	Vegetable soup	20.5	*17.5*, *24.8*	33.7	*29.5*, *39.6*
	Artichoke chicken	41.2	*34.6*, *48.7*	50.0	*43.2*, *57.7*
	Eggplant *parmigiana*	27.8	*25.6*, *32.4*	37.2	*33.8*, *42.0*
	Italian fresh food	5.6	*4.9*, *6.5*	38.9	*35.6*, *43.8*
				**CV%** **Kcal/Bite**	**95%CI**
Gender	Female			61.0	*50.9*, *73.0*
	Male			66.6	*56.7*, *74.7*
Cutlery	Fork			59.0	*50.2*, *68.3*
	Spoon			42.7	*37.8*, *49.0*
	Hand			53.9	*48.4*, *60.5*
Meal	Breakfast			58.2	*51.5*, *66.3*
	Lunch			60.6	*53.1*, *68.9*
	Snack			42.1	*39.2*, *46.0*
	Dinner			64.8	*55.4*, *73.4*

**Table 4:** Results from multiple mixed models, stratified by sex. Independent significant covariates for volume, mass, and energy per bite are shown.

	Dependent	Independent Variables	WOMEN				MEN			
			B	SE(B)	*p*	Full Model R^2^	B	SE(B)	*p*	Full Model R^2^
Model 1	Volume	Age (y)	−0.07	0.08	0.32	24%	−0.42	0.09	<0.0001	53%
		Waist (cm)	0.23	0.08	0.002		0.91	0.12	<0.0001	
		Type of Food (random effect)			<0.0001				<0.0001	
Model 2	Mass	Age (y)	−0.09	0.03	0.004	75%	−0.01	0.03	0.59	83%
		VOLUME (mL)	0.46	0.03	<0.0001		0.57	0.03	<0.0001	
		Type of Food (random effect)			<0.0001				<0.0001	
Model 3	Energy	Age (y)	−0.04	0.05	0.31	89%	0.004	0.92	0.92	90%
		VOLUME (mL)	0.12	0.06	0.05		0.25	0.09	0.007	
		MASS (g)	1.74	0.10	<0.0001		1.45	0.13	<0.0001	
		Type of Food (random effect)			<0.0001				<0.0001	
Model 4	Energy	Age (y)	0.06	0.07	0.39	65%	−0.09	0.07	0.20	68%
		VOLUME (mL)	0.08	0.10	0.49		0.41	0.15	0.005	
		MASS (g)	1.61	0.16	<0.0001		1.29	0.19	<0.0001	
		Cutlery (random effect)			<0.0001				<0.0001	
Model 5	Energy	Age (y)	−0.30	0.10	0.004	48%	−0.64	0.13	<0.0001	71%
		Waist (cm)	0.28	0.10	0.008		0.41	0.15	<0.0001	
		Type of Food (random effect)			<0.0001				<0.0001	
Model 6	Energy	Age (y)	−0.30	0.10	0.004	24%	−0.64	0.13	<0.0001	36%
		Waist (cm)	0.28	0.10	0.008		0.41	0.15	<0.0001	
		Cutlery (random effect)			<0.0001				<0.0001	

## Data Availability

The original contributions presented in the study are included in the article, further inquiries can be directed to the corresponding author.

## Abbreviations

The following abbreviations are used in this manuscript:

ANCOVA	Analysis of Covariance
BMI	Body Mass Index
CI	Confidence Interval
CV	Coefficient of Variation
DCTVPH	Department of Cardiac, Thoracic and Vascular Sciences and Public Health
NOTION	measuriNg calOric inTake at populatION level
SD	Standard Deviation
WHO	World Health Organization
